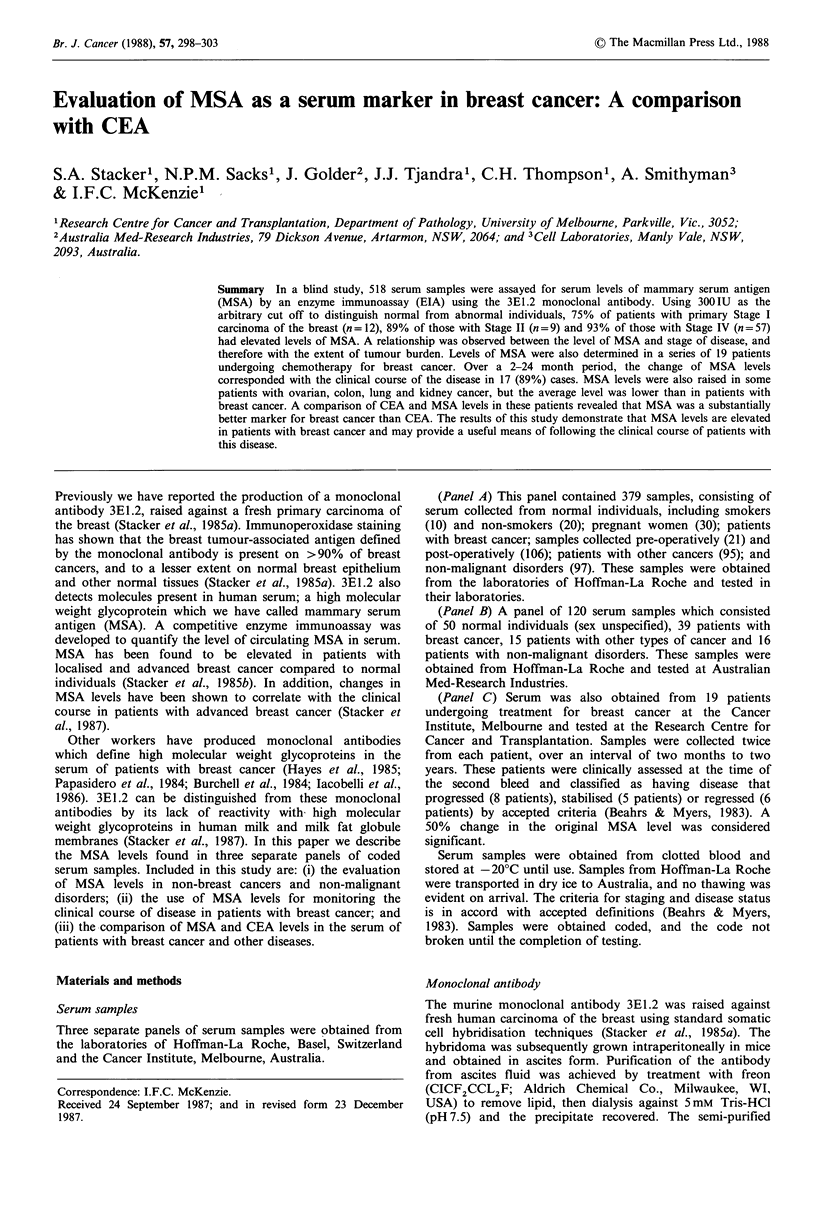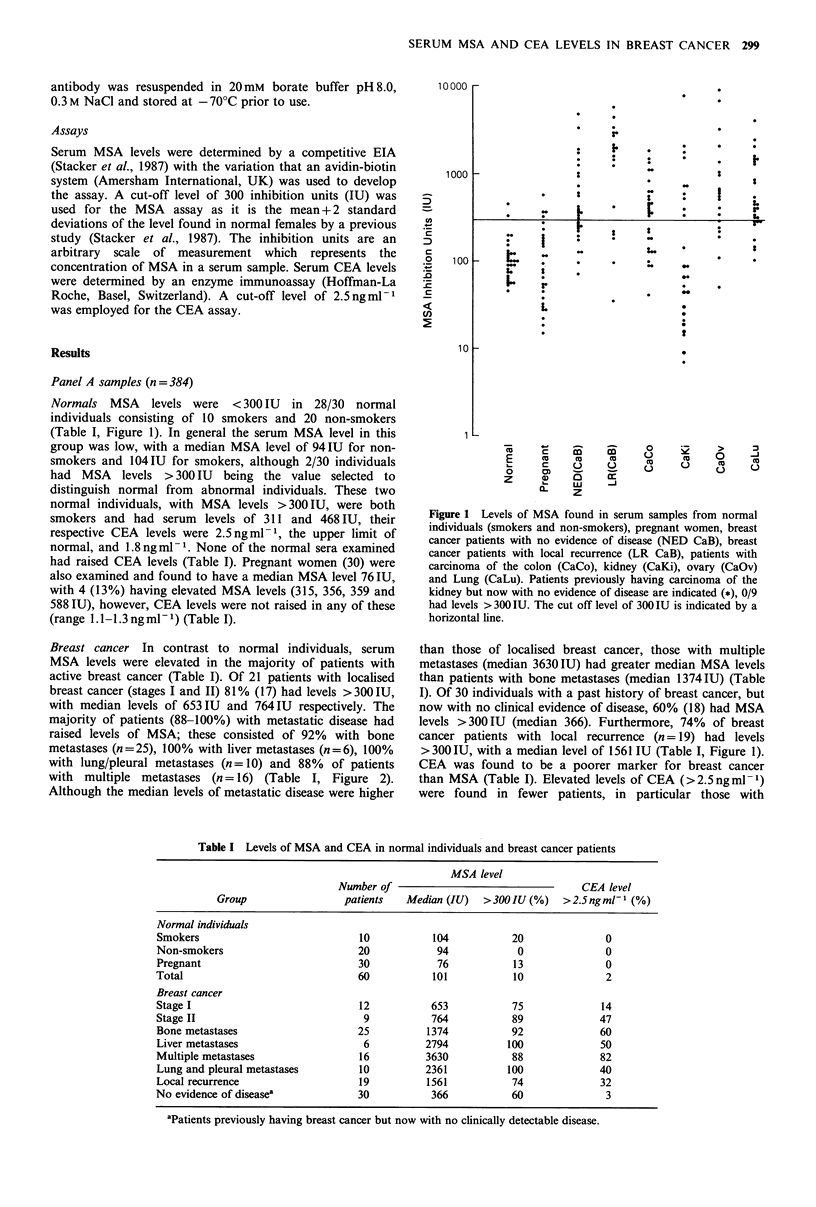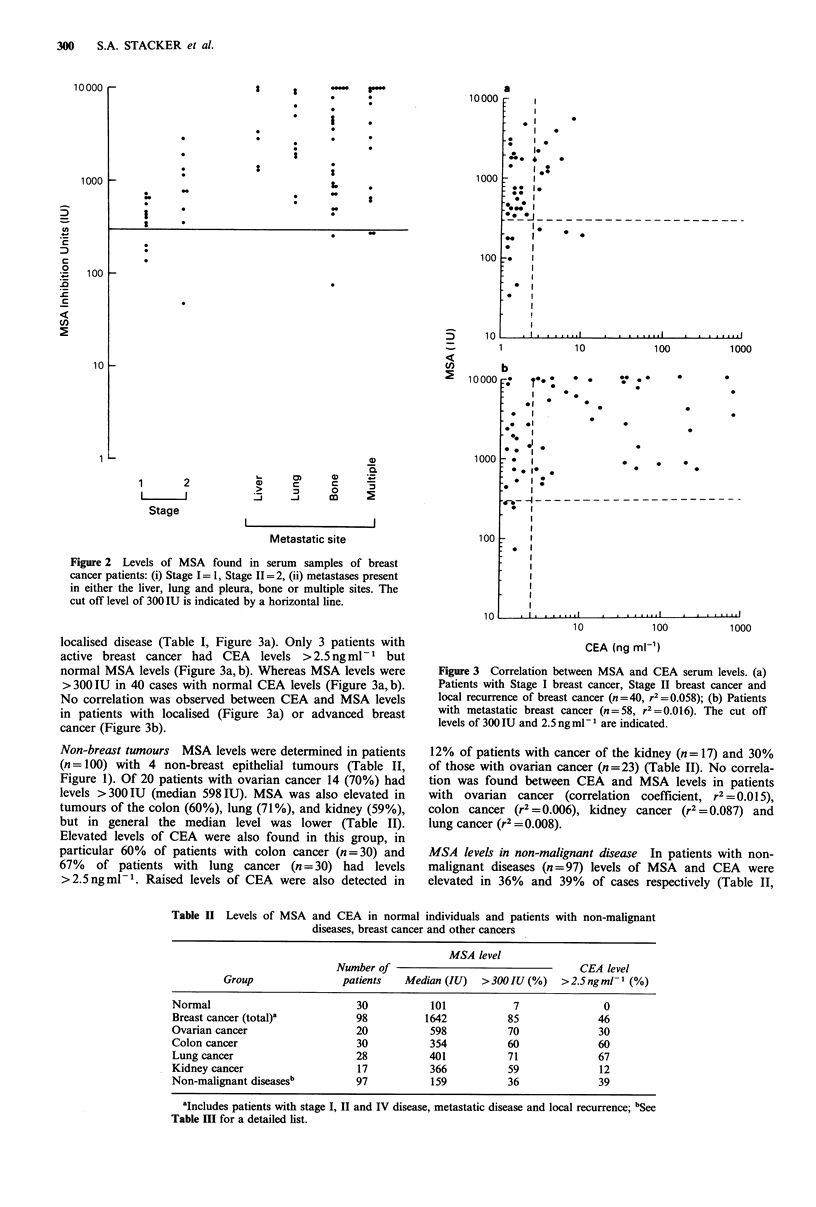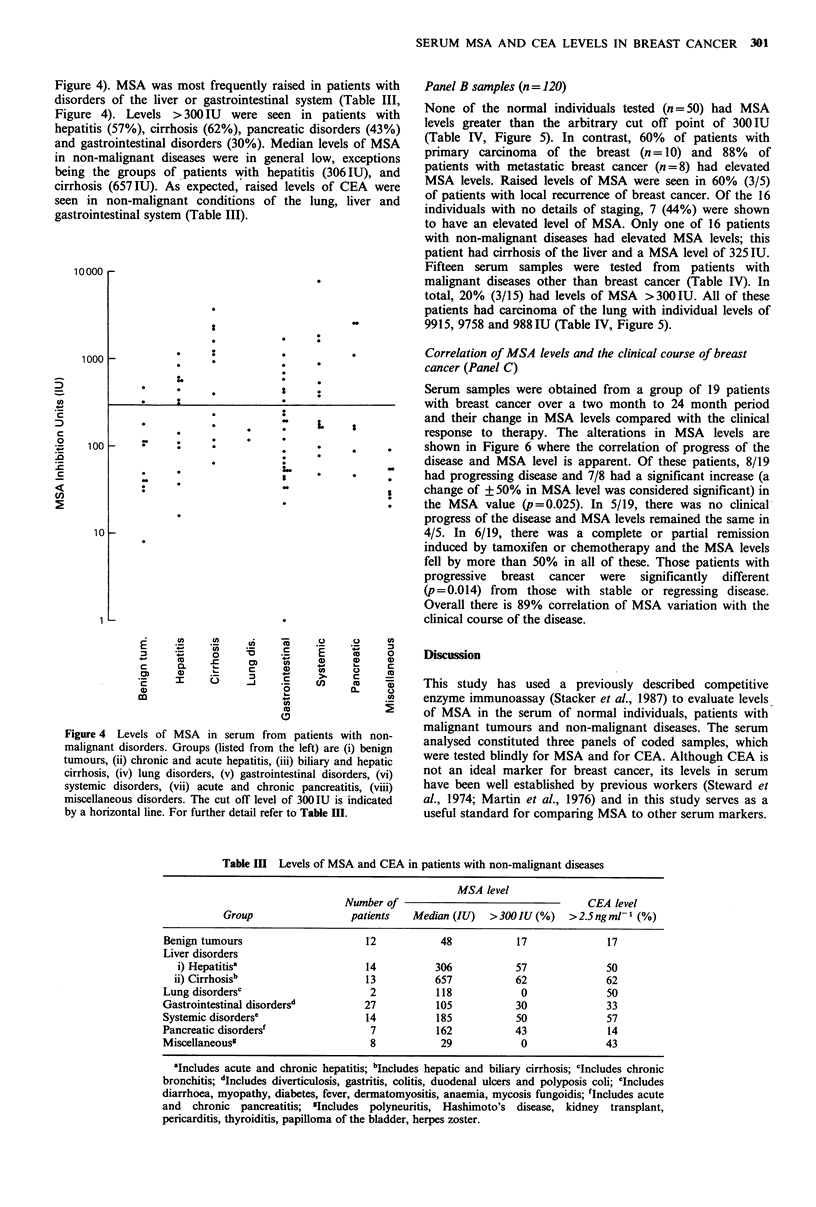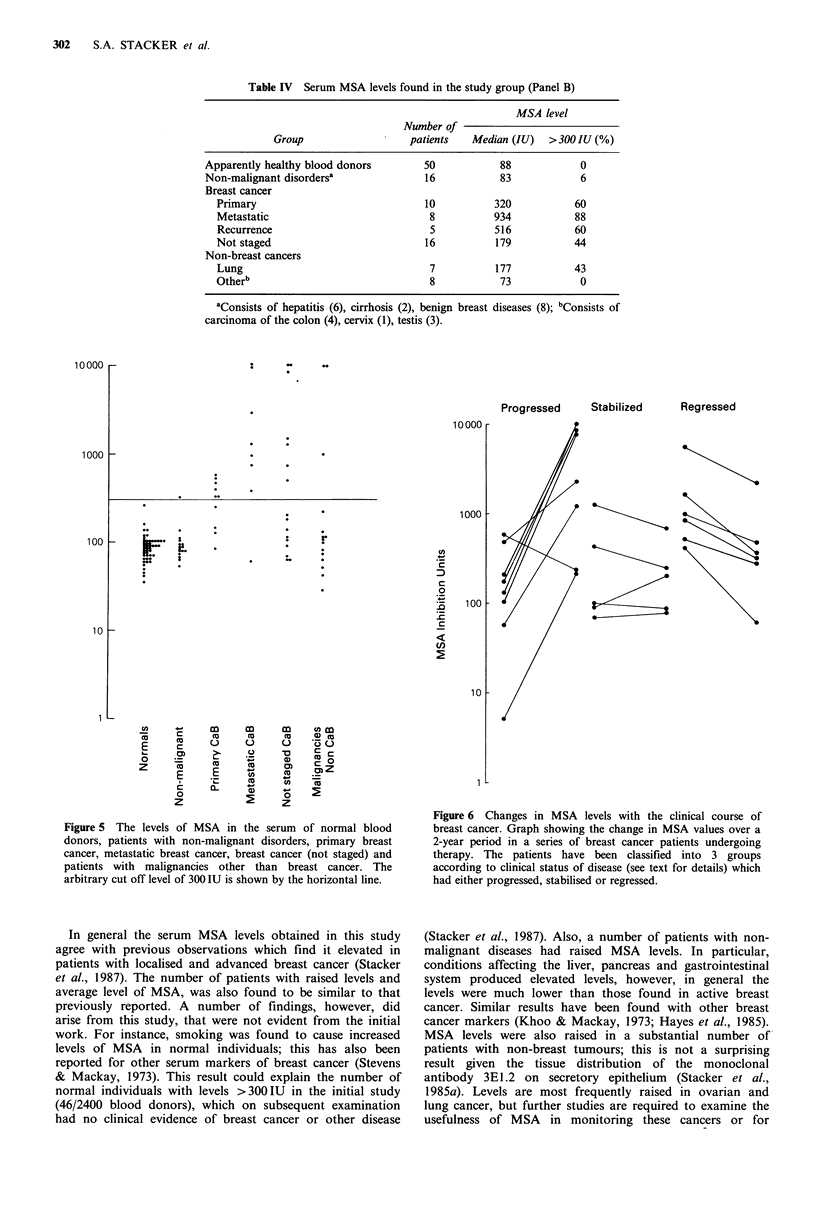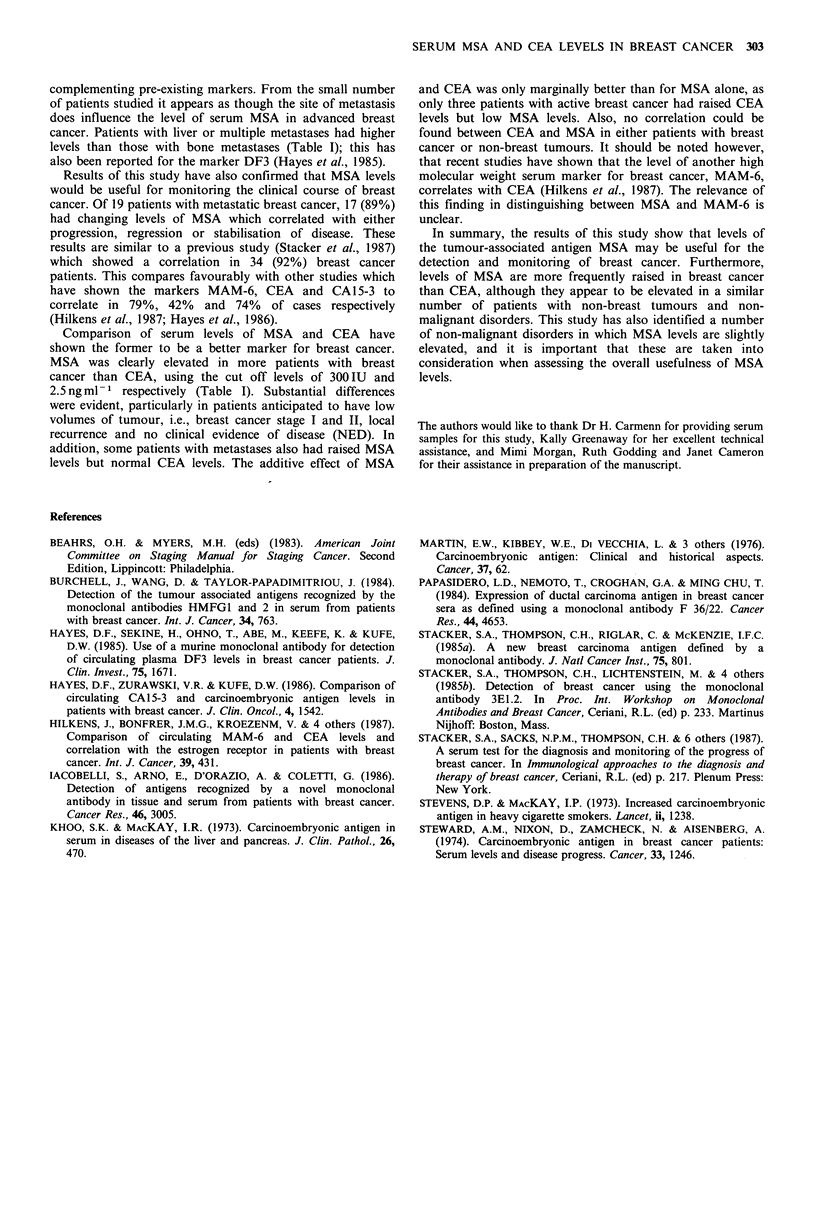# Evaluation of MSA as a serum marker in breast cancer: a comparison with CEA.

**DOI:** 10.1038/bjc.1988.66

**Published:** 1988-03

**Authors:** S. A. Stacker, N. P. Sacks, J. Golder, J. J. Tjandra, C. H. Thompson, A. Smithyman, I. F. McKenzie

**Affiliations:** Department of Pathology, University of Melbourne, Parkville, Vic., Australia.

## Abstract

In a blind study, 518 serum samples were assayed for serum levels of mammary serum antigen (MSA) by an enzyme immunoassay (EIA) using the 3E1.2 monoclonal antibody. Using 300 IU as the arbitrary cut off to distinguish normal from abnormal individuals, 75% of patients with primary Stage I carcinoma of the breast (n = 12), 89% of those with Stage II (n = 9) and 93% of those with Stage IV (n = 57) had elevated levels of MSA. A relationship was observed between the level of MSA and stage of disease, and therefore with the extent of tumour burden. Levels of MSA were also determined in a series of 19 patients undergoing chemotherapy for breast cancer. Over a 2-24 month period, the change of MSA levels corresponded with the clinical course of the disease in 17 (89%) cases. MSA levels were also raised in some patients with ovarian, colon, lung and kidney cancer, but the average level was lower than in patients with breast cancer. A comparison of CEA and MSA levels in these patients revealed that MSA was a substantially better marker for breast cancer than CEA. The results of this study demonstrate that MSA levels are elevated in patients with breast cancer and may provide a useful means of following the clinical course of patients with this disease.


*Br. J. Cancer* (1988), **57**, 298–303 © The Macmillan Press Ltd., 1988

### Evaluation of MSA as a serum marker in breast cancer: A comparison with CEA

S.A. Stacker[1], N.P.M. Sacks[1], J. Golder[2], J.J. Tjandra[1], C.H. Thompson[1], A. Smithyman[3] & I.F.C. McKenzie[1]

[1]*Research Centre for Cancer and Transplantation, Department of Pathology, University of Melbourne, Parkville, Vic., 3052;* [2]*Australia Med-Research Industries, 79 Dickson Avenue, Artarmon, NSW, 2064; and* [3]*Cell Laboratories, Manly Vale, NSW, 2093, Australia.*

**Summary** In a blind study, 518 serum samples were assayed for serum levels of mammary serum antigen (MSA) by an enzyme immunoassay (EIA) using the 3E1.2 monoclonal antibody. Using 300 IU as the arbitrary cut off to distinguish normal from abnormal individuals, 75% of patients with primary Stage I carcinoma of the breast ($n=12$), 89% of those with Stage II ($n=9$) and 93% of those with Stage IV ($n=57$) had elevated levels of MSA. A relationship was observed between the level of MSA and stage of disease, and therefore with the extent of tumour burden. Levels of MSA were also determined in a series of 19 patients undergoing chemotherapy for breast cancer. Over a 2–24 month period, the change of MSA levels corresponded with the clinical course of the disease in 17 (89%) cases. MSA levels were also raised in some patients with ovarian, colon, lung and kidney cancer, but the average level was lower than in patients with breast cancer. A comparison of CEA and MSA levels in these patients revealed that MSA was a substantially better marker for breast cancer than CEA. The results of this study demonstrate that MSA levels are elevated in patients with breast cancer and may provide a useful means of following the clinical course of patients with this disease.

Previously we have reported the production of a monoclonal antibody 3E1.2, raised against a fresh primary carcinoma of the breast (Stacker *et al.*, 1985*a*). Immunoperoxidase staining has shown that the breast tumour-associated antigen defined by the monoclonal antibody is present on >90% of breast cancers, and to a lesser extent on normal breast epithelium and other normal tissues (Stacker *et al.*, 1985*a*). 3E1.2 also detects molecules present in human serum; a high molecular weight glycoprotein which we have called mammary serum antigen (MSA). A competitive enzyme immunoassay was developed to quantify the level of circulating MSA in serum. MSA has been found to be elevated in patients with localised and advanced breast cancer compared to normal individuals (Stacker *et al.*, 1985*b*). In addition, changes in MSA levels have been shown to correlate with the clinical course in patients with advanced breast cancer (Stacker *et al.*, 1987).

Other workers have produced monoclonal antibodies which define high molecular weight glycoproteins in the serum of patients with breast cancer (Hayes *et al.*, 1985; Papasidero *et al.*, 1984; Burchell *et al.*, 1984; Iacobelli *et al.*, 1986). 3E1.2 can be distinguished from these monoclonal antibodies by its lack of reactivity with high molecular weight glycoproteins in human milk and milk fat globule membranes (Stacker *et al.*, 1987). In this paper we describe the MSA levels found in three separate panels of coded serum samples. Included in this study are: (i) the evaluation of MSA levels in non-breast cancers and non-malignant disorders; (ii) the use of MSA levels for monitoring the clinical course of disease in patients with breast cancer; and (iii) the comparison of MSA and CEA levels in the serum of patients with breast cancer and other diseases.

### Materials and methods

### *Serum samples*

Three separate panels of serum samples were obtained from the laboratories of Hoffman-La Roche, Basel, Switzerland and the Cancer Institute, Melbourne, Australia.

Correspondence: I.F.C. McKenzie.
Received 24 September 1987; and in revised form 23 December 1987.

(*Panel A*) This panel contained 379 samples, consisting of serum collected from normal individuals, including smokers (10) and non-smokers (20); pregnant women (30); patients with breast cancer; samples collected pre-operatively (21) and post-operatively (106); patients with other cancers (95); and non-malignant disorders (97). These samples were obtained from the laboratories of Hoffman-La Roche and tested in their laboratories.

(*Panel B*) A panel of 120 serum samples which consisted of 50 normal individuals (sex unspecified), 39 patients with breast cancer, 15 patients with other types of cancer and 16 patients with non-malignant disorders. These samples were obtained from Hoffman-La Roche and tested at Australian Med-Research Industries.

(*Panel C*) Serum was also obtained from 19 patients undergoing treatment for breast cancer at the Cancer Institute, Melbourne and tested at the Research Centre for Cancer and Transplantation. Samples were collected twice from each patient, over an interval of two months to two years. These patients were clinically assessed at the time of the second bleed and classified as having disease that progressed (8 patients), stabilised (5 patients) or regressed (6 patients) by accepted criteria (Beahrs & Myers, 1983). A 50% change in the original MSA level was considered significant.

Serum samples were obtained from clotted blood and stored at −20°C until use. Samples from Hoffman-La Roche were transported in dry ice to Australia, and no thawing was evident on arrival. The criteria for staging and disease status is in accord with accepted definitions (Beahrs & Myers, 1983). Samples were obtained coded, and the code not broken until the completion of testing.

### *Monoclonal antibody*

The murine monoclonal antibody 3E1.2 was raised against fresh human carcinoma of the breast using standard somatic cell hybridisation techniques (Stacker *et al.*, 1985*a*). The hybridoma was subsequently grown intraperitoneally in mice and obtained in ascites form. Purification of the antibody from ascites fluid was achieved by treatment with freon ($ClCF_2CCL_2F$; Aldrich Chemical Co., Milwaukee, WI, USA) to remove lipid, then dialysis against 5 mM Tris-HCl (pH 7.5) and the precipitate recovered. The semi-purified antibody was resuspended in 20 mM borate buffer pH 8.0, 0.3 M NaCl and stored at −70°C prior to use.

*Assays*

Serum MSA levels were determined by a competitive EIA (Stacker *et al.*, 1987) with the variation that an avidin-biotin system (Amersham International, UK) was used to develop the assay. A cut-off level of 300 inhibition units (IU) was used for the MSA assay as it is the mean+2 standard deviations of the level found in normal females by a previous study (Stacker *et al.*, 1987). The inhibition units are an arbitrary scale of measurement which represents the concentration of MSA in a serum sample. Serum CEA levels were determined by an enzyme immunoassay (Hoffman-La Roche, Basel, Switzerland). A cut-off level of 2.5 ng $ml^{-1}$ was employed for the CEA assay.

### Results

*Panel A samples (n = 384)*

*Normals* MSA levels were <300 IU in 28/30 normal individuals consisting of 10 smokers and 20 non-smokers (Table I, Figure 1). In general the serum MSA level in this group was low, with a median MSA level of 94 IU for non-smokers and 104 IU for smokers, although 2/30 individuals had MSA levels >300 IU being the value selected to distinguish normal from abnormal individuals. These two normal individuals, with MSA levels >300 IU, were both smokers and had serum levels of 311 and 468 IU, their respective CEA levels were 2.5 ng $ml^{-1}$, the upper limit of normal, and 1.8 ng $ml^{-1}$. None of the normal sera examined had raised CEA levels (Table I). Pregnant women (30) were also examined and found to have a median MSA level 76 IU, with 4 (13%) having elevated MSA levels (315, 356, 359 and 588 IU), however, CEA levels were not raised in any of these (range 1.1–1.3 ng $ml^{-1}$) (Table I).

*Breast cancer* In contrast to normal individuals, serum MSA levels were elevated in the majority of patients with active breast cancer (Table I). Of 21 patients with localised breast cancer (stages I and II) 81% (17) had levels >300 IU, with median levels of 653 IU and 764 IU respectively. The majority of patients (88–100%) with metastatic disease had raised levels of MSA; these consisted of 92% with bone metastases (n = 25), 100% with liver metastases (n = 6), 100% with lung/pleural metastases (n = 10) and 88% of patients with multiple metastases (n = 16) (Table I, Figure 2). Although the median levels of metastatic disease were higher than those of localised breast cancer, those with multiple metastases (median 3630 IU) had greater median MSA levels than patients with bone metastases (median 1374 IU) (Table I). Of 30 individuals with a past history of breast cancer, but now with no clinical evidence of disease, 60% (18) had MSA levels >300 IU (median 366). Furthermore, 74% of breast cancer patients with local recurrence (n = 19) had levels >300 IU, with a median level of 1561 IU (Table I, Figure 1). CEA was found to be a poorer marker for breast cancer than MSA (Table I). Elevated levels of CEA (>2.5 ng $ml^{-1}$) were found in fewer patients, in particular those with

**Figure 1** Levels of MSA found in serum samples from normal individuals (smokers and non-smokers), pregnant women, breast cancer patients with no evidence of disease (NED CaB), breast cancer patients with local recurrence (LR CaB), patients with carcinoma of the colon (CaCo), kidney (CaKi), ovary (CaOv) and Lung (CaLu). Patients previously having carcinoma of the kidney but now with no evidence of disease are indicated (*), 0/9 had levels >300 IU. The cut off level of 300 IU is indicated by a horizontal line.

**Table I** Levels of MSA and CEA in normal individuals and breast cancer patients

| Group | Number of patients | MSA level Median (IU) | MSA level >300 IU (%) | CEA level >2.5 ng $ml^{-1}$ (%) |
|---|---|---|---|---|
| *Normal individuals* | | | | |
| Smokers | 10 | 104 | 20 | 0 |
| Non-smokers | 20 | 94 | 0 | 0 |
| Pregnant | 30 | 76 | 13 | 0 |
| Total | 60 | 101 | 10 | 2 |
| *Breast cancer* | | | | |
| Stage I | 12 | 653 | 75 | 14 |
| Stage II | 9 | 764 | 89 | 47 |
| Bone metastases | 25 | 1374 | 92 | 60 |
| Liver metastases | 6 | 2794 | 100 | 50 |
| Multiple metastases | 16 | 3630 | 88 | 82 |
| Lung and pleural metastases | 10 | 2361 | 100 | 40 |
| Local recurrence | 19 | 1561 | 74 | 32 |
| No evidence of disease[a] | 30 | 366 | 60 | 3 |

[a]Patients previously having breast cancer but now with no clinically detectable disease.

**Figure 2** Levels of MSA found in serum samples of breast cancer patients: (i) Stage I = 1, Stage II = 2, (ii) metastases present in either the liver, lung and pleura, bone or multiple sites. The cut off level of 300 IU is indicated by a horizontal line.

**Figure 3** Correlation between MSA and CEA serum levels. (a) Patients with Stage I breast cancer, Stage II breast cancer and local recurrence of breast cancer ($n=40$, $r^2=0.058$); (b) Patients with metastatic breast cancer ($n=58$, $r^2=0.016$). The cut off levels of 300 IU and 2.5 ng ml$^{-1}$ are indicated.

localised disease (Table I, Figure 3a). Only 3 patients with active breast cancer had CEA levels >2.5 ng ml$^{-1}$ but normal MSA levels (Figure 3a, b). Whereas MSA levels were >300 IU in 40 cases with normal CEA levels (Figure 3a, b). No correlation was observed between CEA and MSA levels in patients with localised (Figure 3a) or advanced breast cancer (Figure 3b).

*Non-breast tumours* MSA levels were determined in patients ($n=100$) with 4 non-breast epithelial tumours (Table II, Figure 1). Of 20 patients with ovarian cancer 14 (70%) had levels >300 IU (median 598 IU). MSA was also elevated in tumours of the colon (60%), lung (71%), and kidney (59%), but in general the median level was lower (Table II). Elevated levels of CEA were also found in this group, in particular 60% of patients with colon cancer ($n=30$) and 67% of patients with lung cancer ($n=30$) had levels >2.5 ng ml$^{-1}$. Raised levels of CEA were also detected in 12% of patients with cancer of the kidney ($n=17$) and 30% of those with ovarian cancer ($n=23$) (Table II). No correlation was found between CEA and MSA levels in patients with ovarian cancer (correlation coefficient, $r^2=0.015$), colon cancer ($r^2=0.006$), kidney cancer ($r^2=0.087$) and lung cancer ($r^2=0.008$).

*MSA levels in non-malignant disease* In patients with non-malignant diseases ($n=97$) levels of MSA and CEA were elevated in 36% and 39% of cases respectively (Table II,

**Table II** Levels of MSA and CEA in normal individuals and patients with non-malignant diseases, breast cancer and other cancers

| Group | Number of patients | MSA level: Median (IU) | MSA level: >300 IU (%) | CEA level >2.5 ng ml$^{-1}$ (%) |
|---|---|---|---|---|
| Normal | 30 | 101 | 7 | 0 |
| Breast cancer (total)[a] | 98 | 1642 | 85 | 46 |
| Ovarian cancer | 20 | 598 | 70 | 30 |
| Colon cancer | 30 | 354 | 60 | 60 |
| Lung cancer | 28 | 401 | 71 | 67 |
| Kidney cancer | 17 | 366 | 59 | 12 |
| Non-malignant diseases[b] | 97 | 159 | 36 | 39 |

[a]Includes patients with stage I, II and IV disease, metastatic disease and local recurrence; [b]See Table III for a detailed list.

Figure 4). MSA was most frequently raised in patients with disorders of the liver or gastrointestinal system (Table III, Figure 4). Levels >300 IU were seen in patients with hepatitis (57%), cirrhosis (62%), pancreatic disorders (43%) and gastrointestinal disorders (30%). Median levels of MSA in non-malignant diseases were in general low, exceptions being the groups of patients with hepatitis (306 IU), and cirrhosis (657 IU). As expected, raised levels of CEA were seen in non-malignant conditions of the lung, liver and gastrointestinal system (Table III).

**Figure 4** Levels of MSA in serum from patients with non-malignant disorders. Groups (listed from the left) are (i) benign tumours, (ii) chronic and acute hepatitis, (iii) biliary and hepatic cirrhosis, (iv) lung disorders, (v) gastrointestinal disorders, (vi) systemic disorders, (vii) acute and chronic pancreatitis, (viii) miscellaneous disorders. The cut off level of 300 IU is indicated by a horizontal line. For further detail refer to **Table III**.

*Panel B samples* ($n = 120$)

None of the normal individuals tested ($n = 50$) had MSA levels greater than the arbitrary cut off point of 300 IU (Table IV, Figure 5). In contrast, 60% of patients with primary carcinoma of the breast ($n = 10$) and 88% of patients with metastatic breast cancer ($n = 8$) had elevated MSA levels. Raised levels of MSA were seen in 60% (3/5) of patients with local recurrence of breast cancer. Of the 16 individuals with no details of staging, 7 (44%) were shown to have an elevated level of MSA. Only one of 16 patients with non-malignant diseases had elevated MSA levels; this patient had cirrhosis of the liver and a MSA level of 325 IU. Fifteen serum samples were tested from patients with malignant diseases other than breast cancer (Table IV). In total, 20% (3/15) had levels of MSA >300 IU. All of these patients had carcinoma of the lung with individual levels of 9915, 9758 and 988 IU (Table IV, Figure 5).

*Correlation of MSA levels and the clinical course of breast cancer* (*Panel C*)

Serum samples were obtained from a group of 19 patients with breast cancer over a two month to 24 month period and their change in MSA levels compared with the clinical response to therapy. The alterations in MSA levels are shown in Figure 6 where the correlation of progress of the disease and MSA level is apparent. Of these patients, 8/19 had progressing disease and 7/8 had a significant increase (a change of $\pm 50\%$ in MSA level was considered significant) in the MSA value ($p = 0.025$). In 5/19, there was no clinical progress of the disease and MSA levels remained the same in 4/5. In 6/19, there was a complete or partial remission induced by tamoxifen or chemotherapy and the MSA levels fell by more than 50% in all of these. Those patients with progressive breast cancer were significantly different ($p = 0.014$) from those with stable or regressing disease. Overall there is 89% correlation of MSA variation with the clinical course of the disease.

### Discussion

This study has used a previously described competitive enzyme immunoassay (Stacker *et al.*, 1987) to evaluate levels of MSA in the serum of normal individuals, patients with malignant tumours and non-malignant diseases. The serum analysed constituted three panels of coded samples, which were tested blindly for MSA and for CEA. Although CEA is not an ideal marker for breast cancer, its levels in serum have been well established by previous workers (Steward *et al.*, 1974; Martin *et al.*, 1976) and in this study serves as a useful standard for comparing MSA to other serum markers.

**Table III** Levels of MSA and CEA in patients with non-malignant diseases

| *Group* | *Number of patients* | *MSA level* Median (IU) | *MSA level* >300 IU (%) | *CEA level* >2.5 ng ml$^{-1}$ (%) |
|---|---|---|---|---|
| Benign tumours | 12 | 48 | 17 | 17 |
| Liver disorders | | | | |
| i) Hepatitis[a] | 14 | 306 | 57 | 50 |
| ii) Cirrhosis[b] | 13 | 657 | 62 | 62 |
| Lung disorders[c] | 2 | 118 | 0 | 50 |
| Gastrointestinal disorders[d] | 27 | 105 | 30 | 33 |
| Systemic disorders[e] | 14 | 185 | 50 | 57 |
| Pancreatic disorders[f] | 7 | 162 | 43 | 14 |
| Miscellaneous[g] | 8 | 29 | 0 | 43 |

[a]Includes acute and chronic hepatitis; [b]Includes hepatic and biliary cirrhosis; [c]Includes chronic bronchitis; [d]Includes diverticulosis, gastritis, colitis, duodenal ulcers and polyposis coli; [e]Includes diarrhoea, myopathy, diabetes, fever, dermatomyositis, anaemia, mycosis fungoidis; [f]Includes acute and chronic pancreatitis; [g]Includes polyneuritis, Hashimoto's disease, kidney transplant, pericarditis, thyroiditis, papilloma of the bladder, herpes zoster.

**Table IV** Serum MSA levels found in the study group (Panel B)

| *Group* | *Number of patients* | *MSA level* | |
|---|---|---|---|
| | | *Median (IU)* | *>300 IU (%)* |
| Apparently healthy blood donors | 50 | 88 | 0 |
| Non-malignant disorders[a] | 16 | 83 | 6 |
| Breast cancer | | | |
| Primary | 10 | 320 | 60 |
| Metastatic | 8 | 934 | 88 |
| Recurrence | 5 | 516 | 60 |
| Not staged | 16 | 179 | 44 |
| Non-breast cancers | | | |
| Lung | 7 | 177 | 43 |
| Other[b] | 8 | 73 | 0 |

[a]Consists of hepatitis (6), cirrhosis (2), benign breast diseases (8); [b]Consists of carcinoma of the colon (4), cervix (1), testis (3).

**Figure 5** The levels of MSA in the serum of normal blood donors, patients with non-malignant disorders, primary breast cancer, metastatic breast cancer, breast cancer (not staged) and patients with malignancies other than breast cancer. The arbitrary cut off level of 300 IU is shown by the horizontal line.

**Figure 6** Changes in MSA levels with the clinical course of breast cancer. Graph showing the change in MSA values over a 2-year period in a series of breast cancer patients undergoing therapy. The patients have been classified into 3 groups according to clinical status of disease (see text for details) which had either progressed, stabilised or regressed.

In general the serum MSA levels obtained in this study agree with previous observations which find it elevated in patients with localised and advanced breast cancer (Stacker *et al.*, 1987). The number of patients with raised levels and average level of MSA, was also found to be similar to that previously reported. A number of findings, however, did arise from this study, that were not evident from the initial work. For instance, smoking was found to cause increased levels of MSA in normal individuals; this has also been reported for other serum markers of breast cancer (Stevens & Mackay, 1973). This result could explain the number of normal individuals with levels >300 IU in the initial study (46/2400 blood donors), which on subsequent examination had no clinical evidence of breast cancer or other disease (Stacker *et al.*, 1987). Also, a number of patients with non-malignant diseases had raised MSA levels. In particular, conditions affecting the liver, pancreas and gastrointestinal system produced elevated levels, however, in general the levels were much lower than those found in active breast cancer. Similar results have been found with other breast cancer markers (Khoo & Mackay, 1973; Hayes *et al.*, 1985). MSA levels were also raised in a substantial number of patients with non-breast tumours; this is not a surprising result given the tissue distribution of the monoclonal antibody 3E1.2 on secretory epithelium (Stacker *et al.*, 1985*a*). Levels are most frequently raised in ovarian and lung cancer, but further studies are required to examine the usefulness of MSA in monitoring these cancers or for complementing pre-existing markers. From the small number of patients studied it appears as though the site of metastasis does influence the level of serum MSA in advanced breast cancer. Patients with liver or multiple metastases had higher levels than those with bone metastases (Table I); this has also been reported for the marker DF3 (Hayes *et al.*, 1985).

Results of this study have also confirmed that MSA levels would be useful for monitoring the clinical course of breast cancer. Of 19 patients with metastatic breast cancer, 17 (89%) had changing levels of MSA which correlated with either progression, regression or stabilisation of disease. These results are similar to a previous study (Stacker *et al.*, 1987) which showed a correlation in 34 (92%) breast cancer patients. This compares favourably with other studies which have shown the markers MAM-6, CEA and CA15-3 to correlate in 79%, 42% and 74% of cases respectively (Hilkens *et al.*, 1987; Hayes *et al.*, 1986).

Comparison of serum levels of MSA and CEA have shown the former to be a better marker for breast cancer. MSA was clearly elevated in more patients with breast cancer than CEA, using the cut off levels of 300 IU and 2.5 ng ml$^{-1}$ respectively (Table I). Substantial differences were evident, particularly in patients anticipated to have low volumes of tumour, i.e., breast cancer stage I and II, local recurrence and no clinical evidence of disease (NED). In addition, some patients with metastases also had raised MSA levels but normal CEA levels. The additive effect of MSA and CEA was only marginally better than for MSA alone, as only three patients with active breast cancer had raised CEA levels but low MSA levels. Also, no correlation could be found between CEA and MSA in either patients with breast cancer or non-breast tumours. It should be noted however, that recent studies have shown that the level of another high molecular weight serum marker for breast cancer, MAM-6, correlates with CEA (Hilkens *et al.*, 1987). The relevance of this finding in distinguishing between MSA and MAM-6 is unclear.

In summary, the results of this study show that levels of the tumour-associated antigen MSA may be useful for the detection and monitoring of breast cancer. Furthermore, levels of MSA are more frequently raised in breast cancer than CEA, although they appear to be elevated in a similar number of patients with non-breast tumours and non-malignant disorders. This study has also identified a number of non-malignant disorders in which MSA levels are slightly elevated, and it is important that these are taken into consideration when assessing the overall usefulness of MSA levels.

The authors would like to thank Dr H. Carmenn for providing serum samples for this study, Kally Greenaway for her excellent technical assistance, and Mimi Morgan, Ruth Godding and Janet Cameron for their assistance in preparation of the manuscript.

### References

BEAHRS, O.H. & MYERS, M.H. (eds) (1983). *American Joint Committee on Staging Manual for Staging Cancer*. Second Edition, Lippincott: Philadelphia.

BURCHELL, J., WANG, D. & TAYLOR-PAPADIMITRIOU, J. (1984). Detection of the tumour associated antigens recognized by the monoclonal antibodies HMFG1 and 2 in serum from patients with breast cancer. *Int. J. Cancer*, **34**, 763.

HAYES, D.F., SEKINE, H., OHNO, T., ABE, M., KEEFE, K. & KUFE, D.W. (1985). Use of a murine monoclonal antibody for detection of circulating plasma DF3 levels in breast cancer patients. *J. Clin. Invest.*, **75**, 1671.

HAYES, D.F., ZURAWSKI, V.R. & KUFE, D.W. (1986). Comparison of circulating CA15-3 and carcinoembryonic antigen levels in patients with breast cancer. *J. Clin. Oncol.*, **4**, 1542.

HILKENS, J., BONFRER, J.M.G., KROEZENM, V. & 4 others (1987). Comparison of circulating MAM-6 and CEA levels and correlation with the estrogen receptor in patients with breast cancer. *Int. J. Cancer*, **39**, 431.

IACOBELLI, S., ARNO, E., D'ORAZIO, A. & COLETTI, G. (1986). Detection of antigens recognized by a novel monoclonal antibody in tissue and serum from patients with breast cancer. *Cancer Res.*, **46**, 3005.

KHOO, S.K. & MACKAY, I.R. (1973). Carcinoembryonic antigen in serum in diseases of the liver and pancreas. *J. Clin. Pathol.*, **26**, 470.

MARTIN, E.W., KIBBEY, W.E., DI VECCHIA, L. & 3 others (1976). Carcinoembryonic antigen: Clinical and historical aspects. *Cancer*, **37**, 62.

PAPASIDERO, L.D., NEMOTO, T., CROGHAN, G.A. & MING CHU, T. (1984). Expression of ductal carcinoma antigen in breast cancer sera as defined using a monoclonal antibody F 36/22. *Cancer Res.*, **44**, 4653.

STACKER, S.A., THOMPSON, C.H., RIGLAR, C. & MCKENZIE, I.F.C. (1985*a*). A new breast carcinoma antigen defined by a monoclonal antibody. *J. Natl Cancer Inst.*, **75**, 801.

STACKER, S.A., THOMPSON, C.H., LICHTENSTEIN, M. & 4 others (1985*b*). Detection of breast cancer using the monoclonal antibody 3E1.2. In *Proc. Int. Workshop on Monoclonal Antibodies and Breast Cancer*, Ceriani, R.L. (ed) p. 233. Martinus Nijhoff: Boston, Mass.

STACKER, S.A., SACKS, N.P.M., THOMPSON, C.H. & 6 others (1987). A serum test for the diagnosis and monitoring of the progress of breast cancer. In *Immunological approaches to the diagnosis and therapy of breast cancer*, Ceriani, R.L. (ed) p. 217. Plenum Press: New York.

STEVENS, D.P. & MACKAY, I.P. (1973). Increased carcinoembryonic antigen in heavy cigarette smokers. *Lancet*, **ii**, 1238.

STEWARD, A.M., NIXON, D., ZAMCHECK, N. & AISENBERG, A. (1974). Carcinoembryonic antigen in breast cancer patients: Serum levels and disease progress. *Cancer*, **33**, 1246.